# Editorial: Are there different types of child-directed speech? Dynamic variations according to individual and contextual factors

**DOI:** 10.3389/fpsyg.2022.1056816

**Published:** 2023-01-24

**Authors:** Maria Spinelli, Chiara Suttora, Adrian Garcia-Sierra, Fabia Franco, Francesca Lionetti, Mirco Fasolo

**Affiliations:** ^1^Department of Neurosciences, Imaging and Clinical Sciences, University of Studies G. d'Annunzio Chieti and Pescara, Chieti, Italy; ^2^Department of Psychology, University of Bologna, Bologna, Italy; ^3^Department of Speech, Language and Hearing Sciences, University of Connecticut, Storrs, CT, United States; ^4^Department of Psychology, Middlesex University, London, United Kingdom

**Keywords:** child directed speech, caregiver-child interaction, child development, language development, cognitive development, emotional development

Child-directed speech (CDS) is the particular voice register observed in the majority of caregivers during interaction with their infants and children. CDS represents a crucial part of the dyadic caregiver-child interaction, and its prosodic, lexical, syntactic, and functional characteristics are associated with several outcomes such as infant attention, engagement, linguistic acquisition, and affect transmission and sharing (Fernald and Kuhl, 1987; Hoff and Naigles, 2002; Saint-Georges et al., 2013; Rowe and Snow, 2020).

From a dynamic perspective, the specific features and modifications over time of CDS may be considered a way caregivers adjust their input to children's development and achievements during interactions (Soderstrom, 2007; Golinkoff et al., 2015). It is through these social exchanges that, in turn, CDS stimulates children's socio-cognitive development. However, individual fluctuations in CDS characteristics are documented (D'Odorico and Jacob, 2006). These variations—which are not necessarily adaptive or non-adaptive—could be determined by the dynamic interaction among contextual and individual factors.

The current Research Topic brings together researchers working on exploring CDS from this perspective by analyzing specific linguistic and prosodic characteristics of CDS, how these are affected by individual, dyadic, and contextual factors, and the role of those variations in child development.

The importance of considering CDS by focusing on its role in the dyadic exchanges is transversal to all the studies collected in this Research Topic. When exploring CDS universality across cultures and contexts (see Soderstrom et al. and Sarvasy et al.), the findings evidence that even if some elements of CDS appear similar across different languages and cultural contexts, each language has its specific characteristics. These peculiarities appear to be related to the peculiarities of the caregiver-child interaction, which can be influenced by the caregiver's cultural background and practices.

Other studies underlined the importance of considering the role of the child in dyadic exchanges. Fetus-directed speech is influenced by fetal movements, interpreted by mothers as the participation of the fetus in the social interaction (see Parlato-Oliveira et al.). This reciprocal influence is not generic, but specific to the different characteristics of CDS. At 12 months, during interactive turns, the caregiver's IDS phonetic complexity negatively influences the infant's vocalization (see Marklund et al.). The age of the child is a relevant variable in this process. The 10 studies covered different child developmental ages, from fetal to school age, showing how the important characteristics of CDS to explore are different, as are the different competencies and needs of the child. Studies with different time points confirmed that CDS varied over infancy and childhood (see Gram Garmann et al.), adapting to the infant's and children's communication abilities. These variations play an important role in later language development in children (see Cychosz et al.).

Child language development was shown to be influenced by the ability of the caregiver to adapt CDS to the contingent needs of the child over time. For example, maternal circumstances (i.e., parenting stress), dyad aspects (i.e., quality of co-regulation) as well as infant specific circumstances (i.e., preterm birth; see Spinelli et al.). Again, CDS varies according to the specific characteristics of the actors in the dyadic interaction. These variabilities are not always interpreted as different from normative CDS, but as specific adaptations of maternal speech to the infant's needs and communication abilities. Adaptations can be more or less appropriate according to the characteristics of the dyad. It is within environmental and individual risk contexts that the last two studies in this Research Topic illustrate the characteristics and effectiveness of interventions aimed at improving caregivers' CDS to promote, in turn, children's linguistic development (see Hindman et al. and Suttora et al.).

These 10 studies together positively answer the main question of this Research Topic: Are there different types of CDS? CDS was proved to be a dynamic construct, not stable, with variations in the function of the dynamic needs of the dyadic interaction. From this point of view, when studying CDS, the individual role of the child as an interactive partner should be always considered, from the first fetus-caregiver to the teacher/caregiver in interactions with school-aged children. To this extent, going beyond the communicative role of the child (i.e., considering linguistic comprehension and production abilities), researchers should take into account the complexity of the child as an individual with all his/her emotional, interactive, and cognitive abilities. At the same time, the adult should be considered in their complexity as an interactive partner, who uses CDS as an interactive modality with the aim not only to transmit language but also to communicate emotions, affect, and knowledge. Through this lens, considering the caregiver's individual characteristics, such as wellbeing and culture becomes necessary. All these elements dynamically influence each other in the dyadic interactive experience.

This point of view also affects how we should study the impact of CDS on child development. The multifarious peculiarities of CDS imply that CDS might have multifarious purposes, presuming it affects not only a child's linguistic competencies but also several other developmental aspects. We highly encourage more longitudinal studies on this point. It is with such studies that scientific knowledge could gain more information to develop suitable intervention programs to promote the best child development.

In conclusion, the present Research Topic successfully collected contributions from researchers and clinicians, evidencing the importance of looking at CDS from a dynamic perspective and considering the interaction of individual and contextual factors. This is possible only if there is a virtuous integration of expertise between linguistic, emotional, cognitive, and clinical frameworks. Planning studies within this perspective is a future challenge that will advance scientific knowledge in directions that could strongly affect our ability to provide novel knowledge on CDS and hence shape efficient preventive and intervention strategies to promote its quality and its impact on child development. The present Research Topic is the first step forward in this direction.

## Author contributions

All authors listed have made a substantial, direct, and intellectual contribution to the work and approved it for publication.
